# Exploring the Potential Molecular Targets of Cyanidin-3-O-glucoside for Type 2 Diabetes Mellitus Treatment

**DOI:** 10.2174/0118715303421032260216104543

**Published:** 2026-04-08

**Authors:** Wenling Yang, Meizhen Wu, Juanlie Luo, Yaohua Li, Xiaojiao Pan, Hui Tian

**Affiliations:** 1 College of Pharmacy, Guangxi University of Chinese Medicine, Nanning 530200, China;; 2 Guangxi Engineering Research Center of Ethnic Medicine Resources and Application, Nanning 530200, China;; 3 Key Laboratory of TCM Extraction and Purification and Quality Analysis, Nanning 530200, China

**Keywords:** Cyanidin-3-O-glucoside, type 2 diabetes mellitus, potential targets, signaling pathways, molecular docking, molecular dynamics simulation

## Abstract

**Introduction:**

This study aims to elucidate the multi-target molecular mechanism of cyanidin-3-O-glucoside (C3G) in treating Type 2 Diabetes (T2DM) through network pharmacology methods.

**Methods:**

The study was designed to predict the targets of C3G through public databases and to screen for T2DM-related targets. Protein-protein interaction (PPI) network analysis, Gene Ontology (GO), and Kyoto Encyclopedia of Genes and Genomes (KEGG) enrichment analysis were performed on the common targets. Core targets were further validated through molecular docking and molecular dynamics (MD) simulations.

**Results:**

This research identified a total of 57 potential targets of C3G in the treatment of T2DM. Subsequent PPI analysis identified ALB (Degree=43), AKT1 (Degree=41), and TNF (Degree=41) as the top three hub proteins. Pathway analysis indicated significant involvement in the insulin signaling pathway (*P* = 4.205×10^−9^), AMPK signaling pathway (*P* = 9.582×10^−7^), and FoxO signaling pathway (*P* = 1.315×10^−6^). Molecular docking revealed strong binding affinities between C3G and NOS3 (-9.5 kcal/mol), PPARG (-9.0 kcal/mol), TNF (-8.5 kcal/mol), and INSR (-8.4 kcal/mol). MD simulations further confirmed that the C3G-target complex has excellent binding stability.

**Discussion:**

C3G may intervene in the pathological progression of T2DM by regulating key pathways such as insulin sensitivity, inflammatory responses, and oxidative stress. Further studies suggest that INSR and NOS3 may be new targets through which C3G exerts its effects, but their specific mechanisms and *in vivo* biological functions still need to be elucidated by subsequent experiments.

**Conclusion:**

C3G may intervene in the progression of T2DM in a multi-pathway synergistic manner by targeting key molecules such as INSR and NOS3.

## 1 INTRODUCTION

Type 2 Diabetes Mellitus (T2DM) is a chronic metabolic disease primarily characterized by insulin resistance and impaired pancreatic β-cell function. It is widespread globally and poses an increasingly severe public health challenge [1, 2]. According to the International Diabetes Federation (IDF), the number of T2DM patients worldwide reached 537 million in 2021 and is projected to rise to 783 million by 2045 [3]. The harm caused by this disease goes far beyond abnormal blood glucose levels; it is a major trigger for a variety of serious complications, including cardiovascular disease, diabetic nephropathy, and retinopathy. Its high rates of disability and mortality impose a heavy burden on social development [4-7].

Currently, the clinical treatment of type 2 diabetes mainly relies on insulin and oral hypoglycemic drugs [8-10]. However, these conventional therapies have obvious limitations [11]: insulin may cause hypoglycemia and weight gain [12-14], while commonly used oral drugs such as metformin and sulfonylureas can easily lead to gastrointestinal disturbances, and long-term use may carry risks of organ toxicity or reduced efficacy [15-18]. The drawbacks brought about by this single-target mode of action not only affect patients' quality of life but also limit long-term treatment effectiveness. Therefore, finding natural compounds that combine high safety with multi-target synergistic effects has become an important research direction in this field.

Given the limitations of existing therapies, phytochemicals have become a key focus in the development of new drugs for T2DM. These substances cannot only simultaneously regulate multiple interconnected pathological pathways but also have relatively good safety profiles, thus attracting widespread attention from researchers [19]. Cyanidin-3-O-glucoside (C3G) is a common anthocyanin widely found in blueberries, grapes, and black rice, and has been shown to exhibit various biological activities such as antioxidant, anti-inflammatory, and anti-tumor effects, and improvement of glucose and lipid metabolism disorders [20-23]. Evidence from animal models suggests that C3G can enhance insulin sensitivity, protect β-cell function, and improve glucose and lipid homeostasis [24-28]. However, the specific targets and molecular mechanisms of C3G in treating T2DM have not been fully elucidated, particularly the regulatory mechanisms of C3G on the core pathways of insulin resistance have not been systematically studied.

This study aims to systematically investigate the potential targets and mechanisms of C3G in the treatment of T2DM through network pharmacology, molecular docking, and molecular dynamics simulation. By aggregating C3G and T2DM-related targets from multiple databases, we constructed a C3G-T2DM target-pathway network and performed Gene Ontology (GO) and Kyoto Encyclopedia of Genes and Genomes (KEGG) enrichment analyses to identify key targets, biological processes, and signaling pathways. The study findings provide novel theoretical insights and potential directions for the clinical application of C3G in T2DM treatment. The workflow of this study is illustrated in Fig. (**1**).

## 2 MATERIALS AND METHODS

### 2.1 Prediction of C3G Targets

Potential C3G targets were predicted using the SwissTargetPrediction database [29] (http://www.swisstargetprediction.ch), which identifies protein targets based on 2D/3D chemical structure similarity. Additionally, the TCMSP database [30] (http://lsp.nwu.edu.cn/tcmsp.php) was utilized to analyze drug-target-disease associations.

The CAS number of C3G was entered into TCMSP, and its molecular structure was saved in “Mol2” format. This file was subsequently uploaded to PharmMapper [31] (http://lilab.ecust.edu.cn/pharmmapper) for reverse pharmacophore matching. Parameter settings were as follows: Generate Conformers: Yes; Maximum Generated Conformations: 300; Select Target: Human Protein Targets Only (v2010, 2241 entries); Number of Reserved Matched Targets: 300. The predicted targets from SwissTargetPrediction and the top 30 targets from PharmMapper were standardized using the UniProt database (https://www.uniprot.org). Redundant entries were eliminated to generate the final list of C3G targets.

### 2.2 Retrieval of T2DM-Related Targets

T2DM-associated targets were retrieved from the following databases: the Therapeutic Target Database (TTD) [32] (https://db.idrblab.org), Online Mendelian Inheritance in Man (OMIM) [33] (https://www.omim.org), and GeneCards (http://www.genecards.org) [34].

The keyword “Type 2 Diabetes Mellitus” was used for target retrieval, and only targets with scores above the median were included. R-edundant entries were removed, and target names were standardized *via* UniProt [35].

### 2.3 Identification of C3G Anti-Diabetic Targets

The overlapping targets between C3G and T2DM were identified using the Venny v2.1.0 tool (http://bioinfogp.cnb.csic.es/tools/venny/) [36]. These intersection targets were considered potential therapeutic targets for C3G in T2DM.

### 2.4 Protein-Protein Interaction (PPI) Network Construction and Analysis

The overlapping targets were imported into the STRING database [37] (http://string-db.org) for PPI network analysis. The following parameters were applied: Organism: Homo sapiens; Minimum Interaction Score: Medium confidence (0.400); Disconnected nodes were excluded. The PPI network was visualized using Cytoscape 3.10.0 [38], and Network Analyzer was applied to calculate the degree centrality of each node, with higher degrees indicating greater biological relevance.

### 2.5 Pathway Enrichment Analysis

GO functional annotation is categorized into three domains: Biological Process (BP), Cellular Component (CC), and Molecular Function (MF). KEGG pathway analysis [39] serves as a widely adopted bioinformatics resource for functional enrichment and pathway mapping, facilitating the identification of key targets and associated metabolic pathways. GO and KEGG enrichment analyses of the overlapping targets were performed using the clusterProfiler R package (v4.6.0). The Benjamini-Hochberg method was applied for multiple test correction to control the False Discovery Rate (FDR), with a q-value < 0.05 set as the significance threshold. The top 10 significantly enriched terms, ranked by q-value, were visualized using the bioinformatics online platform SRPlot (http://www.bioinformatics.com.cn/srplot) [40].

### 2.6 Molecular Docking

The crystal structures of the target core proteins were obtained from the RCSB Protein Data Bank (http://www.rcsb.org/) [41]. The downloaded protein structures were processed using PyMOL 2.3.0 software [42] to remove original ligands, solvent molecules, and redundant protein chains. The 3D structure of C3G was retrieved from PubChem in SDF format and subsequently optimized using the MMFF94 force field in OpenBabel 3.1.1 to obtain the lowest-energy conformation. The optimized C3G structure was then converted to mol2 format. Prior to docking, hydrogen atoms were added to the proteins using AutoDock Tools 1.5.6 [43], while C3G was prepared by adding hydrogens and assigning rotatable bonds. The prepared protein and ligand structures were exported in the pdbqt format for subsequent molecular docking. The molecular docking parameters were configured using the Grid module, with the specific parameters detailed in Table **1**. Semi-flexible docking was performed with an exhaustiveness value of 25, employing the Lamarckian Genetic Algorithm (LGA) as the search method. Molecular docking was executed using AutoDock Vina 1.2.5 [43], yielding binding free energies and docking pose files for further analysis.

### 2.7 Molecular Dynamics Simulation

In this study, all-atom molecular dynamics simulations were performed on the protein-ligand complexes obtained from molecular docking using the GROMACS 2022.4 software [44]. The protein component was parameterized with the Amber14SB force field [45], while the topology files for the small molecules were generated using the ACPYPE and Antechamber programs [46]. A cubic solvation box was employed, with the shortest distance from the system boundary to the complex set to 1 nm. The TIP3P water model [47] was selected, and an appropriate number of sodium and chloride ions were added to neutralize the system charge.

The V-rescale thermostat was employed to maintain the temperature at 300 K, while the Parrinello-Rahman method was used for pressure control at 101.325 kPa. Subsequently, the system was energy-minimized using the steepest descent method, followed by temperature equilibration in the NVT ensemble and pressure equilibration in the NPT ensemble to stabilize the system at 300 K and 101.325 kPa. For each equilibrated system, a 100 ns molecular dynamics simulation was performed at 300 K [48], yielding 10,000 frames of simulation trajectories. Finally, the obtained trajectory files were used to analyze key parameters such as Root Mean Square Deviation (RMSD), Root Mean Square Fluctuation (RMSF), radius of gyration (Rg), and the total number of hydrogen bonds between the protein and the ligand.

## 3 RESULTS

### 3.1 Target Prediction and Screening

**Fig. (2):**
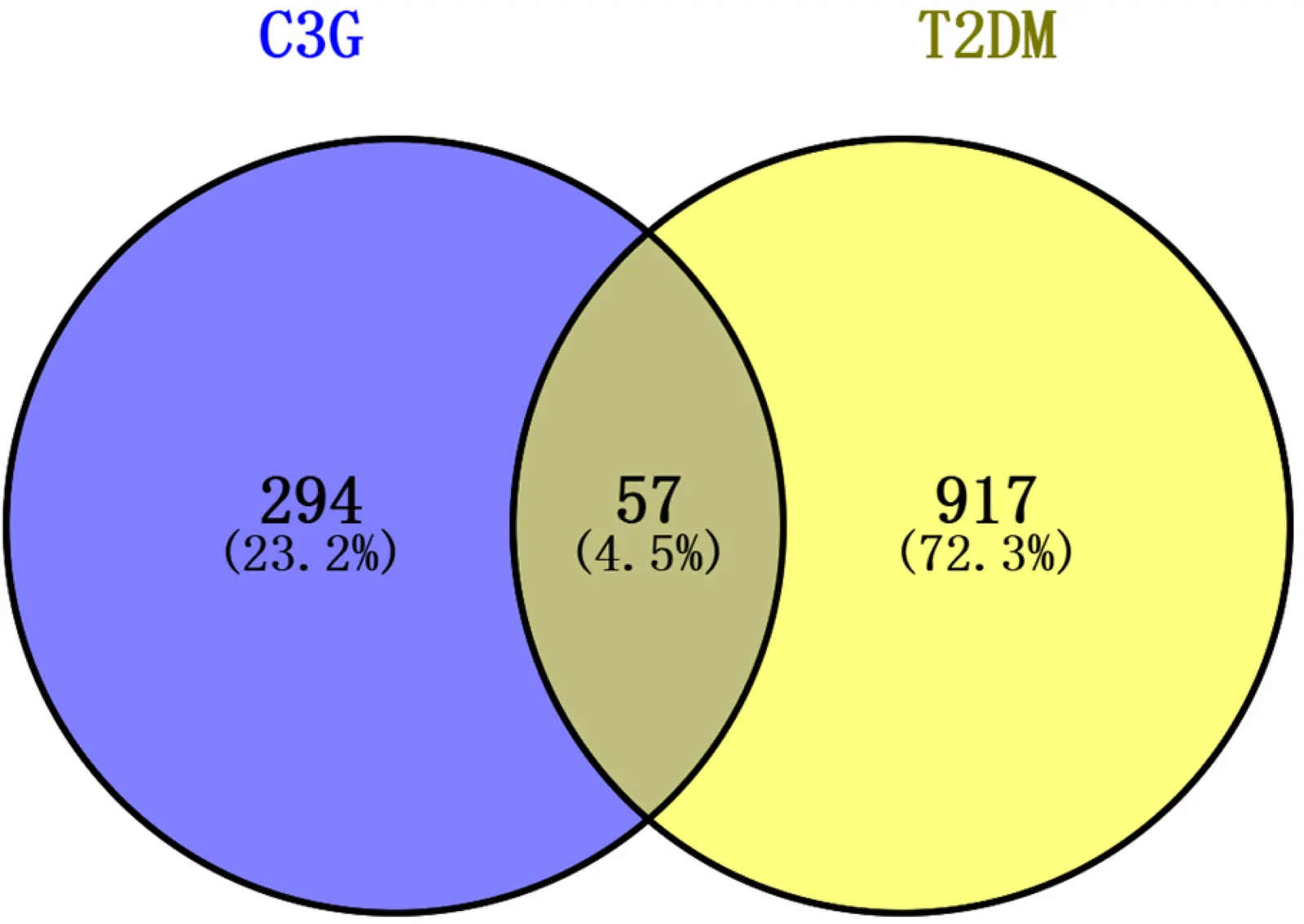
Venn diagram of potential targets of C3G in treating T2DM.

The potential targets of C3G, collected from SwissTargetPrediction and PharmMapper, were screened against a set of 917 T2DM-related targets. This comparison ultimately identified 57 overlapping targets relevant to T2DM treatment (Fig. **2**).

C3G and T2DM-related targets are represented in blue and yellow, respectively, and the overlapping area is identified as the potential targets of C3G for treating T2DM.

### 3.2 PPI Analysis Results

The 57 overlapping genes were further analyzed using the STRING database, and the PPI network was visualized with Cytoscape 3.10.0. As shown in Fig. (**3**), there is a high level of interaction between the targets, with an average node degree of 16.2. Key targets were identified based on their degree values (Table **2**) and categorized them into four groups based on their biological functions: targets related to glucose metabolism and insulin signaling pathway (AKT1, IGF1), targets associated with inflammation and oxidative stress (TNF, PTGS2), targets involved in lipid metabolism regulation (PPARG, PPARA), and targets linked to diabetic complications (ALB, MMP9, HIF1A). The results of this PPI network analysis clearly reveal multiple key potential targets of C3G in the treatment of T2DM and their interactions.

Nodes represent predicted C3G targets, with their size and color intensity proportional to the degree of connectivity. Edges denote functional protein associations. Key hub targets (*e.g.*, ALB, AKT1, TNF, PPARG) are highly interconnected and may be central to the synergistic mechanism of C3G in T2DM treatment.

### 3.3 GO and KEGG Enrichment Analysis Results

GO and KEGG enrichment analyses were conducted using the SRplot platform, generating three types of plots: GO annotation plots, KEGG pathway bubble charts, and cnet plots. The reported significance of the results is based on adjusted *P*-values.

This chart illustrates the GO enrichment analysis of potential C3G targets for T2DM treatment ([adjusted *P*-value < 0.0^5^]). The top 10 significantly enriched terms are color-coded: orange for Biological Process (BP), green for Cellular Component (CC), and blue for Molecular Function (MF).

**Fig. (4):**
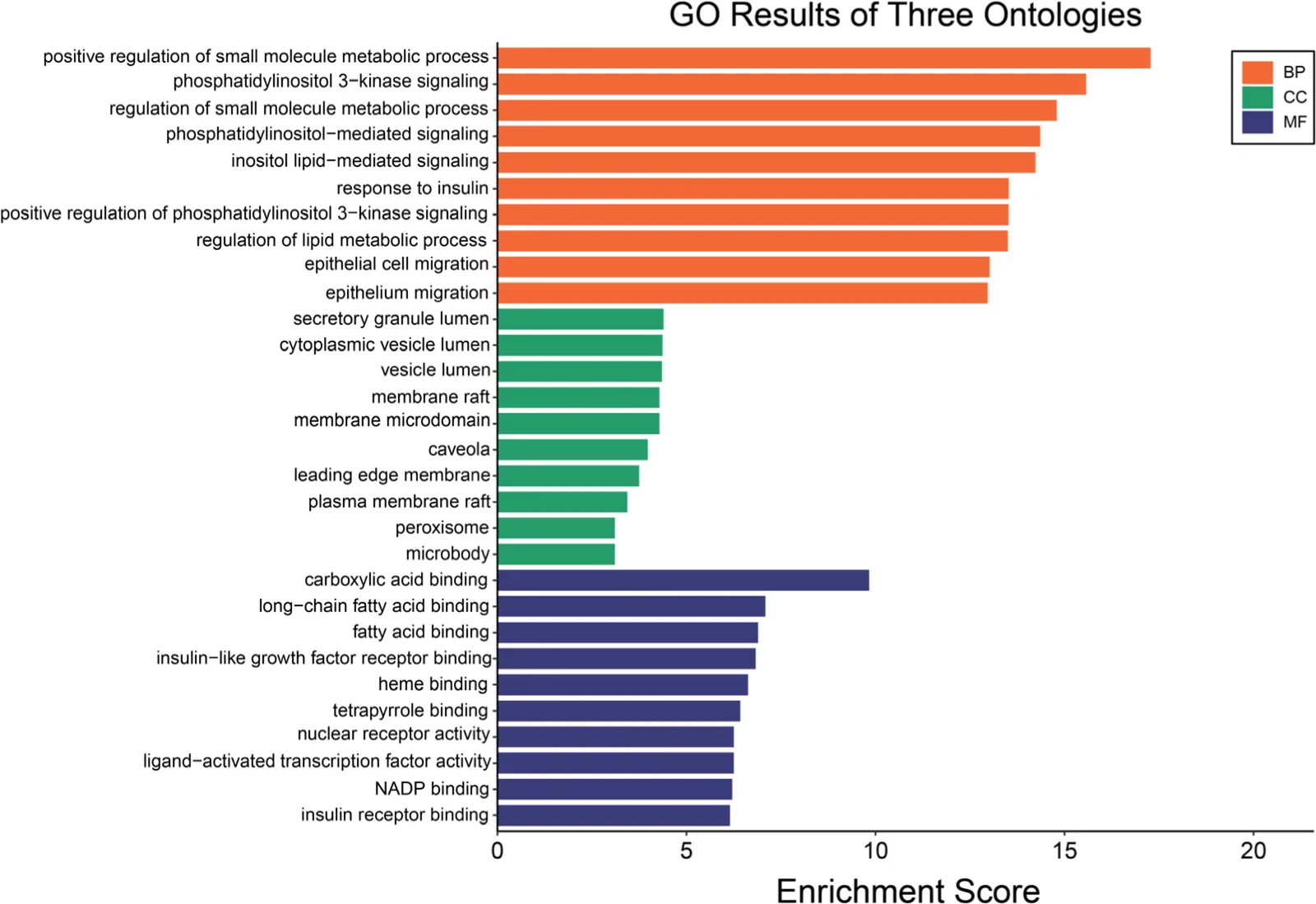
GO functional enrichment analysis of potential targets for C3G in treating T2DM.

BP analysis (Fig. **4**) revealed that the most significantly enriched terms related to T2DM included the following: positive regulation of phosphatidylinositol 3-kinase signaling (*P* = 4.719 × 10^−12^), phosphatidylinositol 3-kinase signaling (*P* = 1.609 × 10^−13^), response to insulin (*P* = 4.719 × 10^−12^), regulation of lipid metabolic process (*P* = 4.719 × 10^−12^), and inositol lipid-mediated signaling (*P* = 1.403 × 10^−12^). As shown in Fig. (**5**), the top overlapping genes in BP analysis included AKT1, TNF, PPARD, PPARG, INSR, FGFR1, PIK3CG, IGF1, CAT, PTGS2, IGF1R, and F2. Collectively, these findings indicate that C3G targets are significantly enriched in biological processes such as PI3K/AKT signaling pathway regulation, inflammation and oxidative stress modulation, and lipid metabolism homeostasis.

BP (Biological Process): The diagram displays the top 10 most significantly enriched terms. Yellow circles represent the enriched terms, with their area sized proportionally to the number of associated genes. Red circles represent the genes. Edges of distinct colors connect each term to its associated genes. In this network, a gene’s degree (*i.e*., its number of connections) reflects how broadly its function overlaps with the enriched biological terms.

**Fig. (6):**
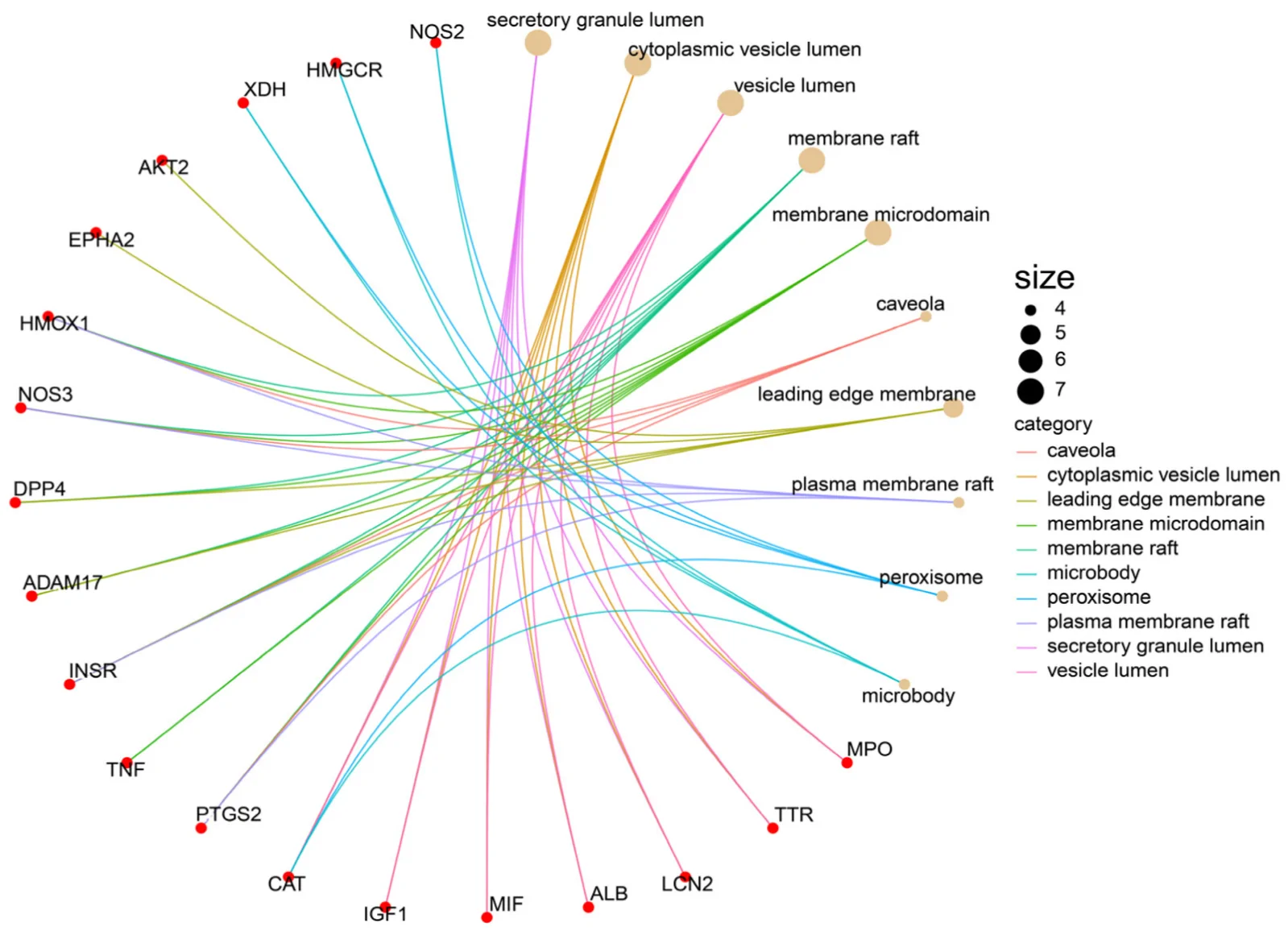
Gene-concept network of significantly enriched CC terms.

CC analysis (Fig. **4**) demonstrated that the most relevant terms associated with T2DM included the following terms: Secretory granule lumen (*P* = 9.674×10^−4^), Membrane raft (*P* = 9.674×10^−4^), Membrane microdomain (*P* = 9.674×10^−4^), Plasma membrane raft (*P* = 4.271×10^−3^), caveola (*P* = 1.644×10^−3^), Peroxisome (*P* = 7.324×10^−3^) and Microbody (*P* = 7.324×10^−3^). The CC analysis (Fig. **6**) revealed key overlapping genes—CAT, INSR, PTGS2, HMOX1, NOS3, ADAM17, and DPP4. C3G targets are mainly distributed within key cellular structures, including the secretory granule lumen, membrane rafts, plasma membrane microdomains, and peroxisomes.

CC (Cellular Component): The diagram illustrates the top 10 most significantly enriched terms. Yellow circles denote these enriched terms, with their area scaled proportionally to the number of linked genes. Red circles correspond to individual genes. Distinctly colored edges link each term to its associated genes. Within this network, a gene’s degree (*i.e.*, its number of connections) indicates the extent of its functional relevance to the enriched biological terms.

**Fig. (7):**
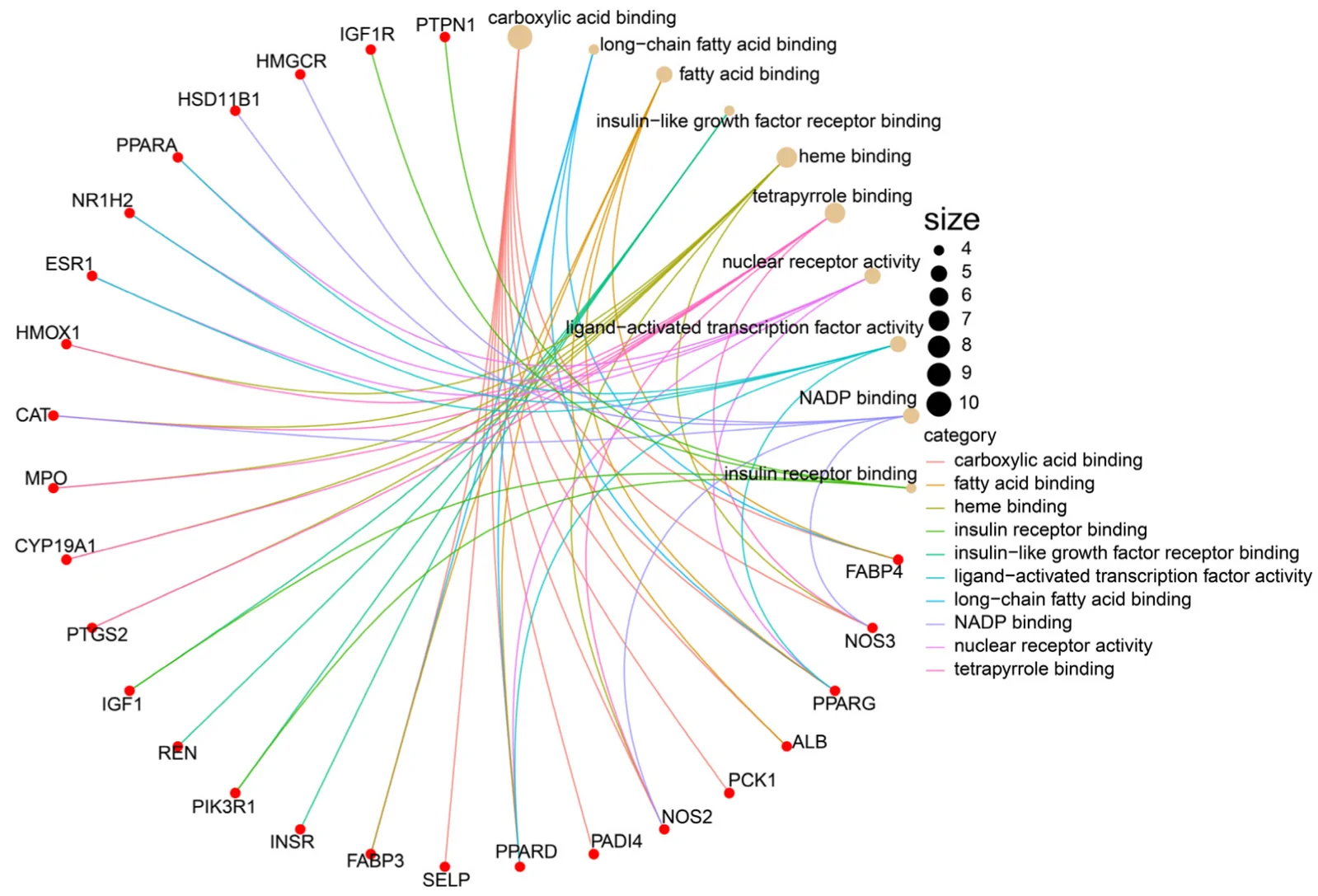
Gene-concept network of significantly enriched MF terms.

MF analysis (Fig. **4**) identified the most critical functional terms, including: insulin receptor binding (*P* = 1.278×10^−5^), Insulin-like growth factor receptor binding (*P* = 6.675×10^−6^), Fatty acid binding (*P* = 6.675×10^−6^), Long-chain fatty acid binding (*P* = 6.675×10^−6^), Nuclear receptor activity (*P* = 1.239×10^−5^), and Ligand-activated transcription factor activity (*P* = 1.239×10^−5^). MF analysis (Fig. **7**) highlighted PPARG, PPARD, NOS3, NOS2, CAT, FABP3, and FABP4 as the top overlapping genes. This result suggests that C3G targets are chiefly associated with key molecular functions, including nuclear receptor activity, insulin receptor binding, and interactions as antioxidant or anti-inflammatory mediators.

MF (Molecular Function): The diagram displays the top 10 most significantly enriched terms. Yellow circles represent the enriched terms, with their area sized proportionally to the number of associated genes. Red circles represent the genes. Edges of distinct colors connect each term to its associated genes. For a given gene, the number of edges connected to it indicates the extent of its overlap among these terms.

**Fig. (8):**
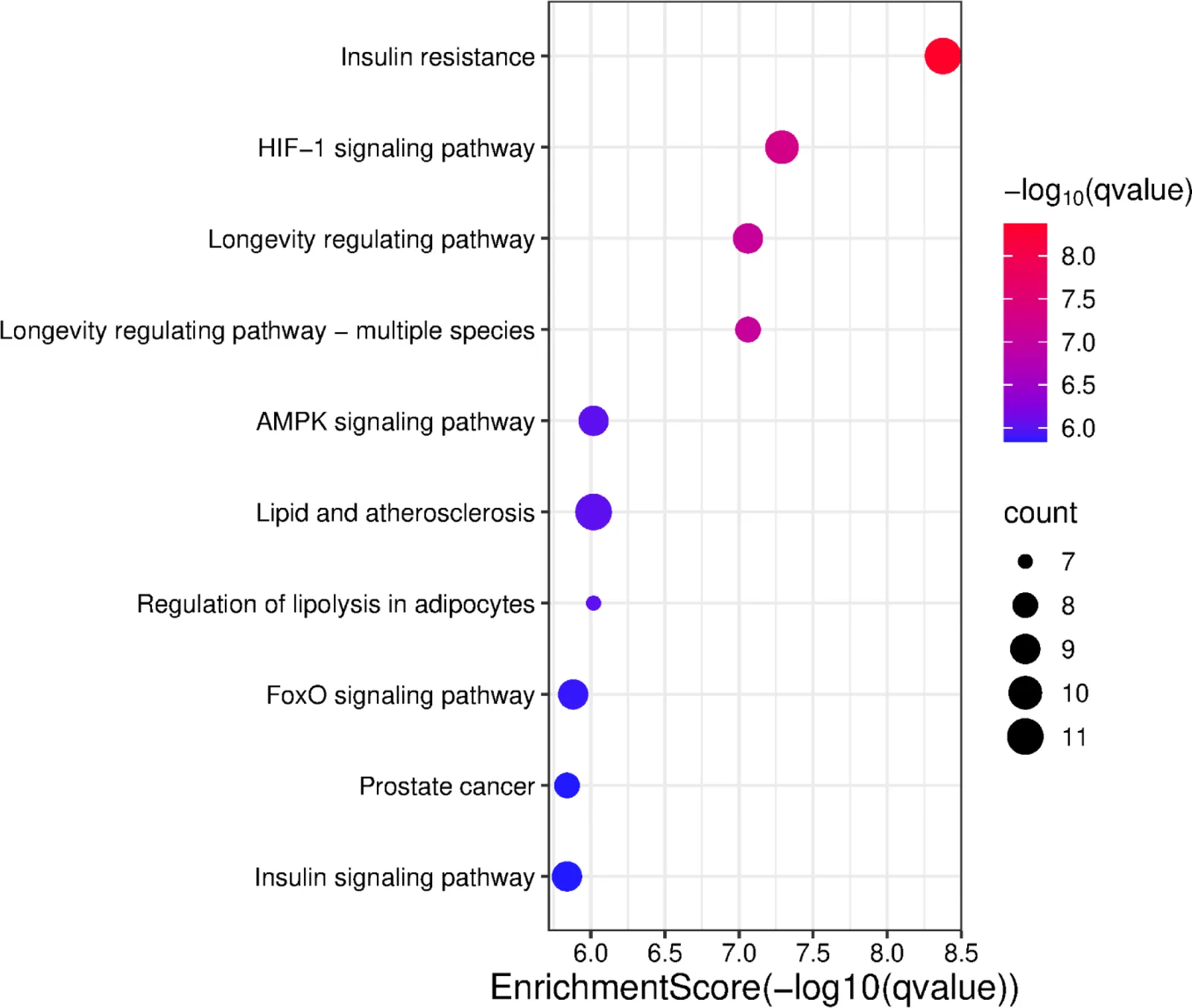
Bubble chart of significantly enriched kegg pathways.

The KEGG pathway analysis (Fig. **8**) identified multiple diabetes-associated pathways, including key pathways such as Insulin resistance (*P* = 4.205×10^−9^), HIF-1 signaling pathway (*P* = 5.134×10^−8^), AMPK signaling pathway (*P* = 9.582×10^−7^), Lipid and atherosclerosis (*P* = 9.582×10^−7^), FoxO signaling pathway (*P* = 1.315×10^−6^), Regulation of lipolysis in adipocytes (*P* = 5.49333×10^−8^)and insulin signaling pathway (*P* = 9.582×10^−7^). As shown in Fig. (**9**), the most prominent overlapping genes in KEGG pathway analysis included AKT1, TNF, INSR, IGF1, PIK3R1, IGF1R, GSK3B, SOD2, PCK1, PPARG, CAT, and NOS3. The potential targets of C3G were significantly enriched in the following pathways: Insulin resistance pathway, HIF-1 signaling pathway, AMPK signaling pathway, and Lipid and atherosclerosis pathway.

The chart displays significantly enriched KEGG pathways. The Y-axis shows the pathway names, while the X-axis represents the number of enriched genes. Bubble size corresponds to the rich factor (the ratio of enriched genes to the total number of genes in a pathway). Bubble color corresponds to the -log10 (q-value), with a color gradient from blue (less significant) to red (more significant), indicating the statistical significance of the enrichment.

The diagram displays the top 10 most significantly enriched terms. Yellow circles represent the enriched terms, with their area sized proportionally to the number of associated genes. Red circles represent the genes. Edges of distinct colors connect each term to its associated genes. For a given gene, the number of edges connected to it indicates the extent of its overlap among these terms.

### 3.4 Molecular Docking Results

**Fig. (10):**
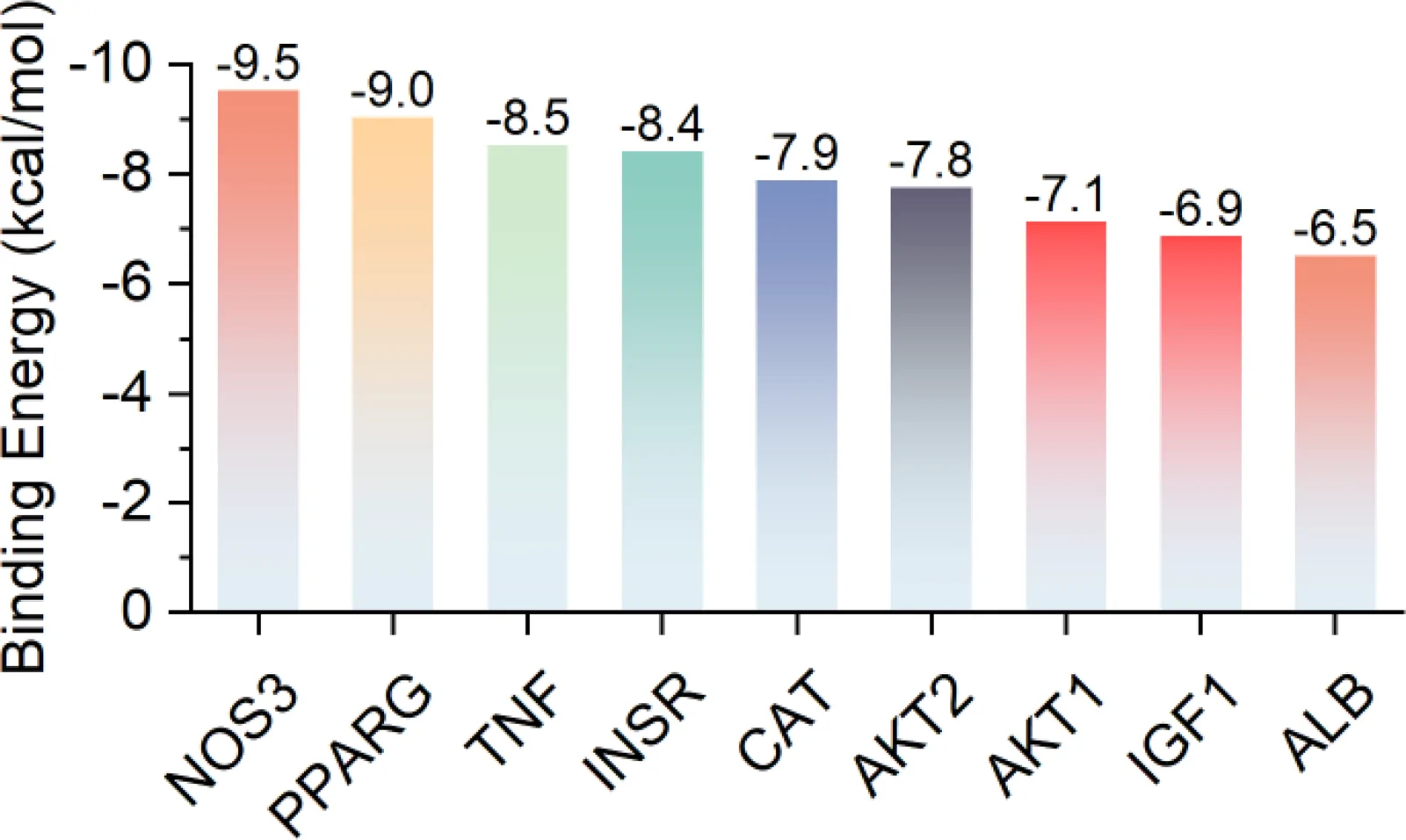
Ranked distribution of molecular docking binding energies for C3G with T2DM-related targets.

Core targets were comprehensively determined by selecting genes with high Degree values from PPI network analysis and those that were significantly enriched and had high intersection frequency in GO and KEGG analyses. These core targets included AKT1, TNF, AKT2, PPARG, IGF1, INSR, NOS3, CAT, and ALB. Molecular docking analysis was performed between C3G and the corresponding proteins of these core targets to calculate their binding energies. A binding energy ≤ -5 kcal/mol indicated excellent binding affinity between C3G and the target. The docking results revealed that the binding energies between C3G and all the aforementioned proteins were below -6.0 kcal/mol (Fig. **10**), with the top four ranking as follows: NOS3 (-9.5 kcal/mol), PPARG (-9.0 kcal/mol), TNF (-8.5 kcal/mol), and INSR (-8.4 kcal/mol). These high-affinity bindings suggest that C3G may directly modulate the functions of these targets, thereby influencing T2DM-related pathways. This finding lays the groundwork for further investigation into the dynamic interactions between C3G and key targets (*e.g.*, INSR, NOS3).

The molecular docking interactions between C3G and these proteins were visualized using PyMOL 2.3.0 software, and the detailed binding modes are illustrated in Fig. (**11**). As illustrated in Fig. (**11**), C3G formed four hydrogen bond interactions with the amino acid residues TRP-356, GLY-355, SER-226, and GLU-361 of NOS3 protein, six hydrogen bond interactions with GLU-343, SER-342, ARG-288, and GLU-295 of PPARG protein, seven hydrogen bond interactions with SER-136, TYR-195, ILE-134, GLY-198, LEU-233, and TYR-227 of TNF protein, and four hydrogen bond interactions with GLU-1077, MET-1079, ALA-1080, and ASP-1083 of INSR protein. Given that all binding energies were below -8 kcal/mol, C3G exhibited strong binding affinity to NOS3, PPARG, TNF, and INSR proteins, suggesting a potentially significant impact on the structural integrity, functional regulation, and biological activity of these proteins. This molecular docking analysis provides robust evidence for the high-affinity binding interactions between C3G and key therapeutic targets (binding energies ≤ -8 kcal/mol), supporting its potential multi-target therapeutic mechanism in T2DM treatment.

This bar chart is arranged in order of predicted binding energy from low to high. The horizontal axis represents the names of the target proteins, and the vertical axis represents the binding free energy calculated by molecular docking (unit: kcal/mol). The lower the binding energy value, the stronger the predicted binding affinity.

### 3.5 Molecular Dynamics Simulation Results

**Fig. (12):**
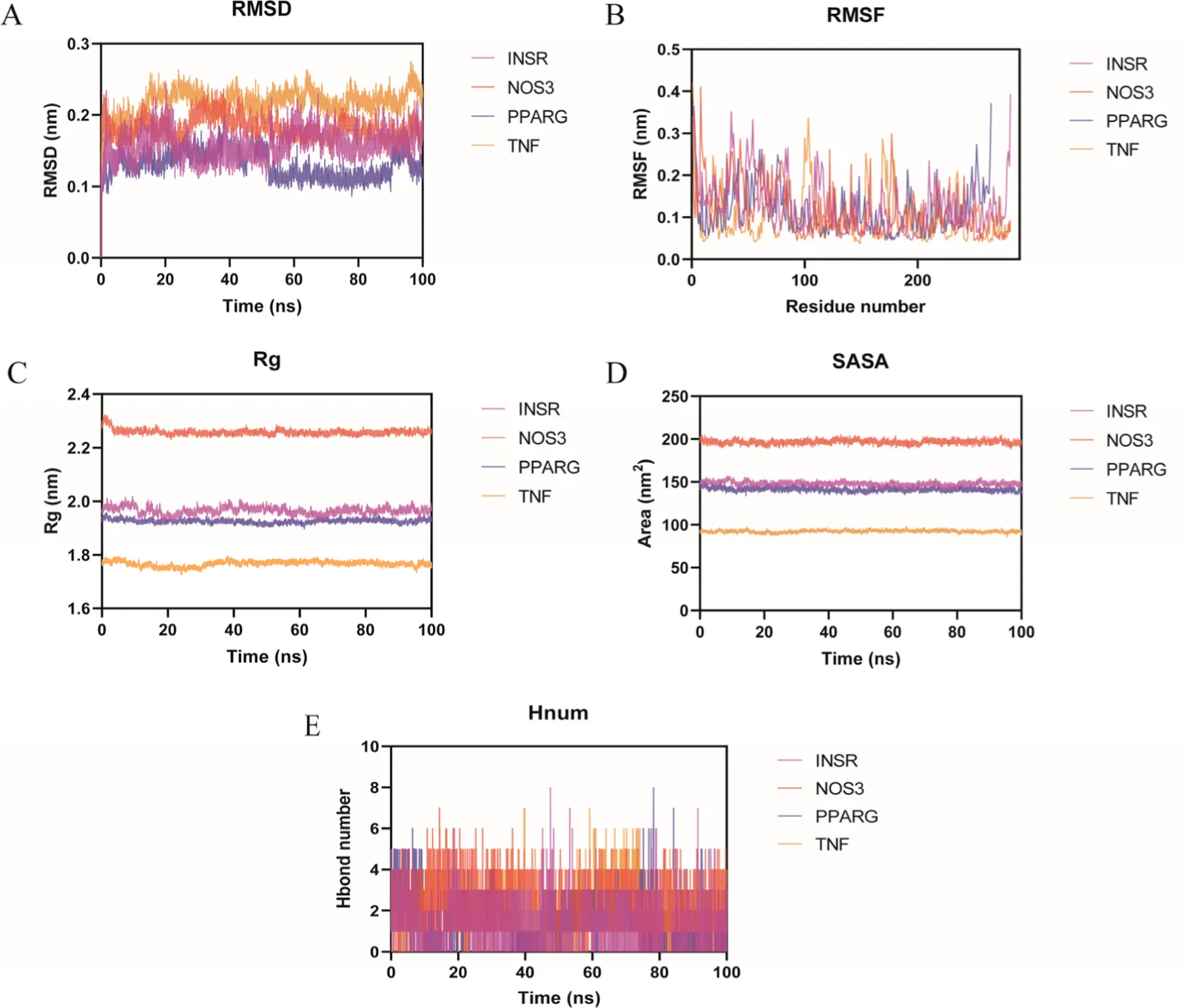
Molecular dynamics of the four target proteins in complex with C3G. Molecular dynamics analyses of the four C3G–protein complexes (distinguished by color). **(A**) Backbone root-mean-square deviation (RMSD) quantifies global conformational stability and system equilibration. (**B**) Per-residue root-mean-square fluctuation (RMSF) maps local flexibility across the protein sequence. (**C**) The radius of gyration (Rg) serves as a metric for the overall structural compactness. (**D**) Solvent-accessible surface area (SASA) reflects changes in protein solvation during the simulation. (**E**) Intermolecular hydrogen-bond counts track the stability of key ligand-protein interactions.

Molecular dynamics simulations confirmed that C3G forms structurally stable complexes with four key target proteins: INSR, NOS3, PPARG, and TNF. Each system reached equilibrium during the mid-to-late stages of the simulation, with key metrics—including conformational stability, local flexibility, overall compactness, and surface properties—converging and mutually corroborating the dynamic stability of the complexes (Fig. **12**).

All complexes attained conformational equilibrium. RMSD analysis revealed that after initial adjustments (0-20 ns), the RMSD values of all systems converged rapidly and remained within narrow ranges (PPARG: ~0.12 nm; INSR: ~0.15 nm; NOS3: ~0.18 nm; TNF: ~0.23 nm). This convergence indicates that C3G binding did not induce persistent conformational drift and that each complex adopted a stable binding mode (Fig. **12A**).

Local and global structural features consistently support stability. First, RMSF analysis showed that residue flexibility in each protein remained within functionally relevant dynamic ranges post-binding, with no abnormal high-flexibility peaks (Fig. **12B**). Second, the evolution of the Rg demonstrated that the overall dimensions of all complexes tightened and stabilized at specific values in the later simulation stages (*e.g*., TNF was the most compact, with Rg ~1.77 nm; PPARG stabilized at 1.93 nm), reflecting a tightly packed and balanced fold state (Fig. **12C**). Finally, solvent-accessible surface area (SASA) remained within relatively stable fluctuation ranges throughout the simulation (*e.g.*, TNF showed the narrowest variation, 90–95 nm^2^), further confirming the overall structural compactness at the protein-solvent interface (Fig. **12D**).

The stability of the complexes was further supported by hydrogen bond analysis. Although some complexes can form up to 7-8 hydrogen bonds simultaneously, throughout the entire simulation, 2-3 key hydrogen bonds were consistently maintained (INSR and PPARG mainly formed 2-3 hydrogen bonds; NOS3 formed 2-4; TNF formed 2-3). The stable network of these key hydrogen bonds is an important factor in maintaining ligand-protein binding stability (Fig. **12E**).

Molecular dynamics simulations confirmed the formation of stable C3G complexes with four target proteins (INSR, NOS3, PPARG, TNF), as demonstrated by the maintenance of structural integrity, restricted backbone fluctuations, and a persistent hydrogen-bond network throughout the simulation.

## 4 DISCUSSION

The therapeutic potential of the natural anthocyanin C3G in targeting core metabolic pathologies, such as insulin resistance, attracts significant research interest. Previous studies indicate that its beneficial effects in T2DM may involve multiple pathways, including the modulation of glucose transporters and adipokines to improve insulin sensitivity [58], as well as binding to PPARG to regulate downstream genes in glycolipid metabolism [27]. Some studies have also indicated that it alleviates inflammation-mediated insulin resistance by inhibiting the production of key cytokine inflammatory factors (such as TNF-α) [59] and inhibiting PTP1B to enhance insulin signaling [26]. Despite these insights, the specific molecular targets of C3G in T2DM and the detailed mechanisms of its interactions with key target proteins remain unclear, particularly the systematic elucidation of target regulation within the core insulin resistance pathways. To systematically elucidate the multi-target mechanisms of C3G in treating T2DM, this study used network pharmacology to predict its potential targets, which were subsequently validated and analyzed in depth through molecular docking and molecular dynamics simulations, examining both static binding modes and dynamic binding stability. The main findings are as follows:

(1) Potent binding and modulation of key targets INSR and NOS3: This study employs integrated computational simulations to demonstrate that C3G engages directly and with high affinity at two critical sites: INSR and NOS3. These interactions collectively offer a unified structural rationale for C3G's established dual role in improving metabolic and vascular health. INSR, a core mediator of insulin signaling, is dysfunctional in the development of T2DM [60]. MD analysis indicates that C3G is likely to stabilize INSR in an active conformational state. This structural stabilization provides a plausible mechanistic basis for the previously confirmed enhancement by C3G of the IRS-1/PI3K/AKT pathway and the subsequent promotion of glucose uptake [61], thereby establishing INSR as a primary target through which it exerts its insulin-sensitizing effects. Notably, C3G shows the strongest predicted binding affinity with NOS3 (-9.5 kcal/mol). In addition to regulating downstream NO bioavailability [62], C3G may act more upstream by directly stabilizing NOS3's own structure. This stabilization could reduce its pathological uncoupling, thereby ensuring sustained physiological NO production. The proposed mechanism addresses two key issues simultaneously: improving endothelium-dependent vascular relaxation and alleviating a critical vascular factor leading to insulin resistance. Therefore, the combined action of C3G with INSR and NOS3 suggests that it may act as a dual-target regulator. This provides a coherent molecular hypothesis for understanding how a single compound can simultaneously improve the characteristic, intertwined metabolic and vascular pathological changes in T2DM.

(2) Target enrichment and synergistic regulation of core pathway insulin resistance pathways: KEGG pathway enrichment analysis showed that the potential targets of C3G were significantly enriched in the insulin resistance pathway (*P* = 4.205×10^−9^). Therefore, this pathway has been established as the core action hub of C3G intervention in T2DM, suggesting that its multi-target effect may be realized through the synergistic regulation of this pathway. Contrary to previous understanding, this study not only confirmed the regulatory effect of C3G on known targets (such as PPARG and TNF), but more importantly, through molecular simulation, it revealed that C3G can directly act with high affinity on the more critical upstream nodes of this pathway - INSR and its important downstream effector, NOS3. This finding is consistent with the viewpoint of Liu *et al*. in their systematic review, that natural products often intervene in complex diseases through multi-target mechanisms [63]. It is believed that synergistic, multi-level regulation from receptor activation to signal transduction and terminal effects is achieved by C3G through its strong binding to INSR and NOS3, thereby providing a new molecular mechanistic basis for understanding its multi-target, multi-pathway therapeutic advantages beyond traditional single-target drugs.

(3) CAT as a direct antioxidant target of C3G: GO analysis and molecular docking both point to CAT as a relevant target of C3G. While C3G's antioxidant effects are well established [64, 65] and often ascribed to direct radical scavenging, docking results show it binds efficiently to CAT (-7.9 kcal/mol). This interaction suggests C3G could directly enhance or stabilize CAT activity, offering a structural explanation for how it mitigates oxidative stress in T2DM. By boosting CAT-mediated clearance of excess H_2_O_2_, C3G may protect insulin signaling components such as IRS-1 from oxidative inactivation. These findings are consistent with reports that C3G elevates antioxidant enzyme activities in diabetic models [66], but go further by identifying CAT as a plausible direct target—effectively linking C3G's non-enzymatic and enzymatic antioxidant modes of action.

(4) The Pivotal Role of AKT1/2 in Insulin Signaling: AKT1 emerged as a central node in the insulin signaling pathway based on PPI network topology and GO/KEGG enrichment analyses. Molecular docking further indicated favorable binding of C3G to both AKT1 and AKT2. While earlier cellular studies have shown C3G-mediated AKT activation [67-69], the present work positions AKT within a multi- target network and provides structural evidence for its direct engagement by C3G. These results reinforce the view that C3G may improve systemic glucose metabolism by coordinating the activation of AKT1—primarily influencing GLUT4 translocation—and AKT2, which regulates hepatic glucose output and contributes to GLUT4 trafficking. Consistently demonstrated in cellular models that C3G effectively activates AKT.

This study also observed a noteworthy phenomenon: ALB, which has the highest connectivity in the PPI network, did not show critical significance in subsequent functional enrichment and molecular docking analyses; meanwhile, INSR and NOS3, which had the best docking binding energies, ranked relatively lower in network connectivity. This seemingly contradictory result precisely suggests that the centrality (degree) in network topology and the functional importance of targets, as well as ligand binding specificity, are two dimensions that need to be evaluated independently.

As the most abundant protein in plasma, ALB's biological function is as a general carrier, responsible for transporting various endogenous substances (such as fatty acids, hormones, and bilirubin) and exogenous substances (such as drugs) [70-72]. It's very high Degree value (43) in the PPI network mainly reflects its extensive physical binding or transport associations, rather than serving as a core regulator in specific signaling pathways. From the molecular docking results, the binding affinity of C3G to ALB (-6.5 kcal/mol) is of moderate strength, significantly lower than its binding forces with core targets such as NOS3, PPARG, TNF, and INSR. Therefore, although ALB may affect the pharmacokinetics (*e.g.*, distribution and half-life) of C3G through nonspecific binding, it is unlikely to be a direct functional target mediating its therapeutic effect. This interaction likely reflects a pharmacokinetic carrier relationship that may influence C3G's distribution, but does not contribute directly to its anti-diabetic pharmacology. Therefore, ALB represents a transport hub in the interactome, not a functional therapeutic target for C3G.

Although INSR and NOS3 have relatively low direct connections (degrees) in PPI networks (not in the top 9), this does not diminish their importance as key functional targets. As the initiator receptor for insulin signaling, INSR's function is specific and irreplaceable. Its activation directly triggers complex downstream signaling cascades (*e.g.*, the IRS/PI3K/AKT pathway) [73], and its lower degree value may reflect a greater focus on interactions with downstream-specific signaling molecules (*e.g.*, IRS1/2) in the network rather than broad, non-specific connections like ALB. The strong binding force revealed by molecular docking simply illustrates C3G's specific and efficient intervention in its function. NOS3, as an enzyme that produces the key signaling molecule NO, is also highly specialized [74]. It may interact primarily with specific molecules in the network that regulate its activity (*e.g.*, *via* phosphorylation) or with its product (NO). Its low degree of value does not affect its status as a core effector molecule for improving endothelial function and insulin sensitivity. The molecular docking results (strongest binding energy) strongly support the potential of C3G to directly and efficiently modulate its function. Although the degree values of INSR and NOS3 did not enter the top 9 PPIs, they were enriched into key pathways (such as insulin signaling pathway, insulin resistance pathway, HIF-1 signaling pathway, *etc*.) in GO/KEGG analysis, which was highly consistent with the strong binding force revealed by molecular docking and the binding stability of molecular dynamics simulations, which together pointed to their core functional role in the mechanism of action of C3G.

The results of network analysis and functional validation revealed fundamental differences in the nature of the targets. Taking ALB as an example, its high network degree stems from its non-specific binding ability as a general carrier, rather than the pathological functions related to diabetes. On the contrary, core targets such as INSR and NOS3, although not having the highest connectivity in the global network, have been confirmed due to their enrichment in related pathways and their high-affinity binding with C3G. Therefore, the multi-target action model of C3G is clarified: its therapeutic effect mainly comes from its high affinity and specificity binding to a few key signaling proteins (INSR, NOS3, PPARG, TNF); while the weaker interactions with proteins like ALB may play an auxiliary role (such as affecting pharmacokinetics). This clear definition of the action mode helps to elucidate the molecular basis for the synergistic efficacy of natural products in complex disease networks like T2DM.

Although this study systematically predicted the potential targets and mechanisms of C3G in treating T2DM, it still has some limitations. First, the network pharmacology analysis draws on existing databases, which could miss certain targets or interactions that have not yet been included. Second, the molecular dynamics simulations were not performed with multiple replicates; future studies should include repeated simulations to better assess the statistical robustness of the results. Furthermore, as the findings are based on computational predictions and simulations—particularly the proposed mechanisms involving INSR and NOS3—they require experimental validation through further *in vitro* (*e.g.*, gene knockout/overexpression, binding assays, signaling detection in cell models) and *in vivo* (*e.g.*, specific gene knockout mouse models) studies. Additionally, this study did not account for the potential influence of tissue-specific expression differences on target function. Future research could leverage single-cell multi-omics technologies to further dissect the mechanism of C3G in specific cell types [75, 76].

## CONCLUSION AND PERSPECTIVES

This study demonstrates that C3G, *via* stable high-affinity binding to key targets, particularly the newly identified strong-binding targets INSR and NOS3, along with known targets (PPARG, TNF, AKT1/2, CAT, and IGF1), synergistically modulates core pathways (insulin resistance, AMPK, FoxO, and HIF-1). By targeting insulin sensitivity, inflammation, and oxidative stress in T2DM, C3G demonstrates a promising multi-target profile. This study identifies potent targets, including INSR and NOS3, providing a theoretical basis for the development of a new generation of insulin sensitizers based on the C3G structure, with both metabolic and vascular protective effects. More importantly, by systematically elucidating the multi-target network of C3G's action on the insulin resistance pathway, this study has laid a solid mechanistic foundation for developing it into a multi-target natural drug or functional food component applicable to the complex pathologies of T2DM. In the future, changes in the activity of these key targets (such as INSR and NOS3), once verified through clinical trials, are expected to become potential biomarkers for evaluating the intervention effect of C3G, thereby establishing a link between mechanism and application.

This study employed computational simulations to reveal the potential mechanism by which C3G acts on key targets such as INSR and NOS3. However, the relevant conclusions require experimental verification. The primary task is to use *in vitro* binding and cell signaling experiments to confirm the direct interaction and functional regulation between C3G and INSR/NOS3, and to evaluate its target-dependent efficacy in diabetic animal models. The low bioavailability of C3G remains a central obstacle to its efficacy and clinical translation [77, 78]. Our finding that C3G binds with high affinity to the vascular key target NOS3 offers a new perspective on this challenge: designing nanodelivery systems capable of targeting the vascular system. This approach represents not only a feasible strategy to improve bioavailability but also defines a prioritized research direction, evaluating the therapeutic potential of C3G in T2DM-related cardiovascular complications. A coherent research pathway thus emerges. Initially, targeted delivery systems can be used to validate the efficacy of C3G against cardiovascular complications in animal models. Subsequently, technologies such as single-cell transcriptomics may be applied to dissect the cell-type-specific response networks in complex tissues (*e.g.*, vasculature, adipose, liver) following C3G treatment. This integrated paradigm—spanning from targeted delivery to cell-resolved mechanistic analysis—will establish a solid foundation for developing precise C3G-based intervention strategies.

## Figures and Tables

**Fig. (1):**
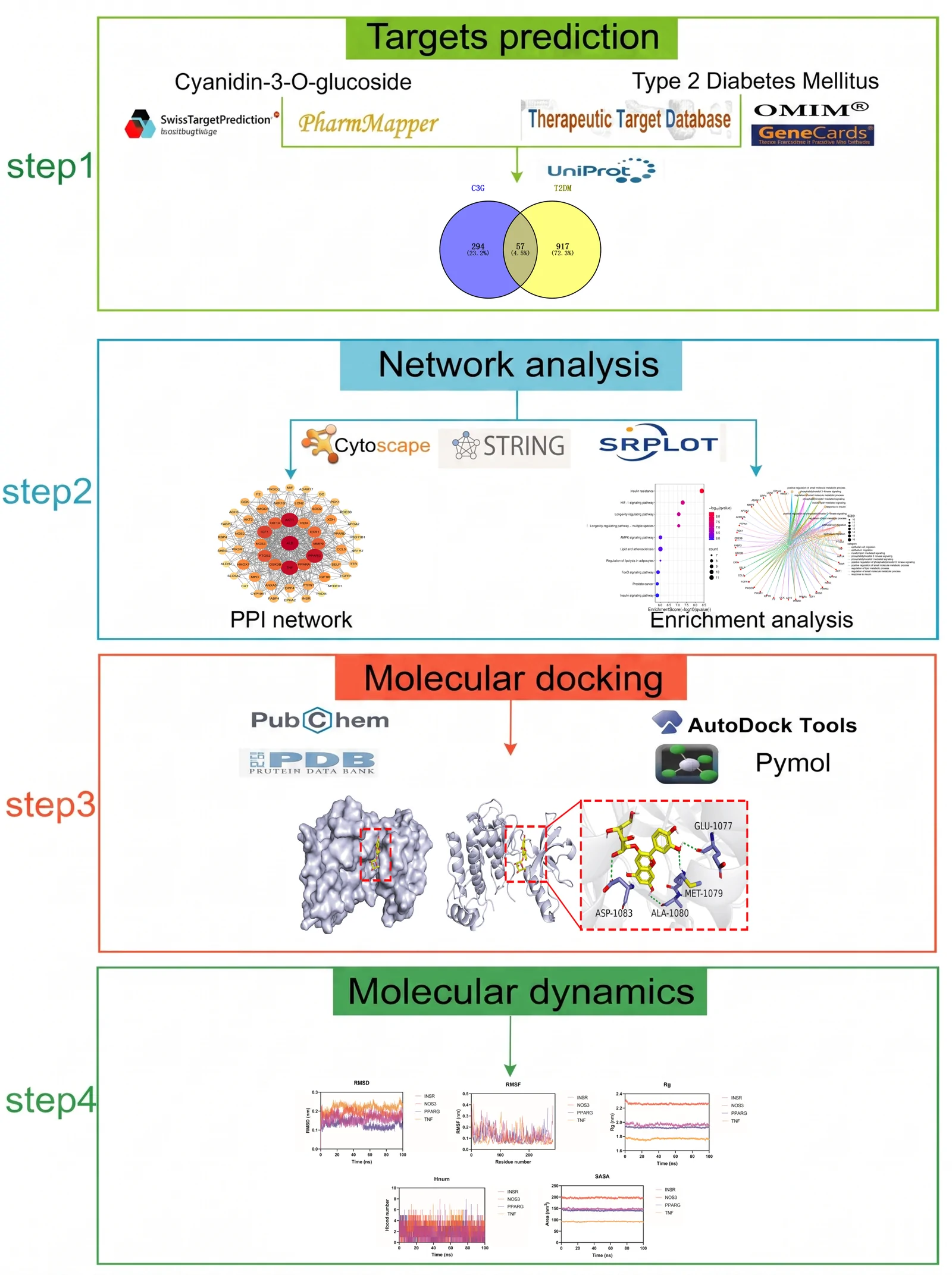
Flowchart of a network pharmacology-based approach to investigate the targets of C3G in the treatment of T2DM.

**Fig. (3):**
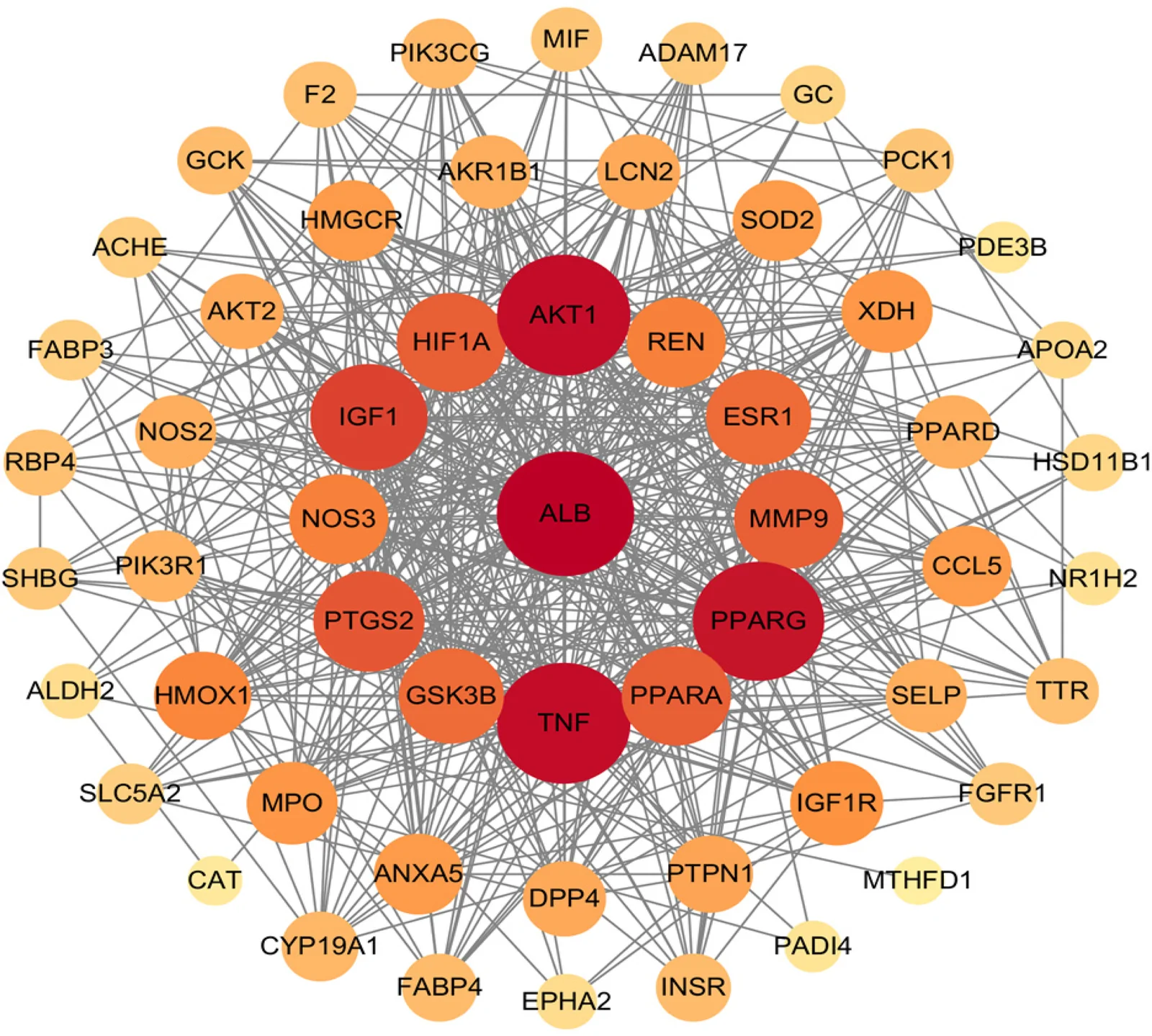
PPI network of C3G targets against T2DM.

**Fig. (5):**
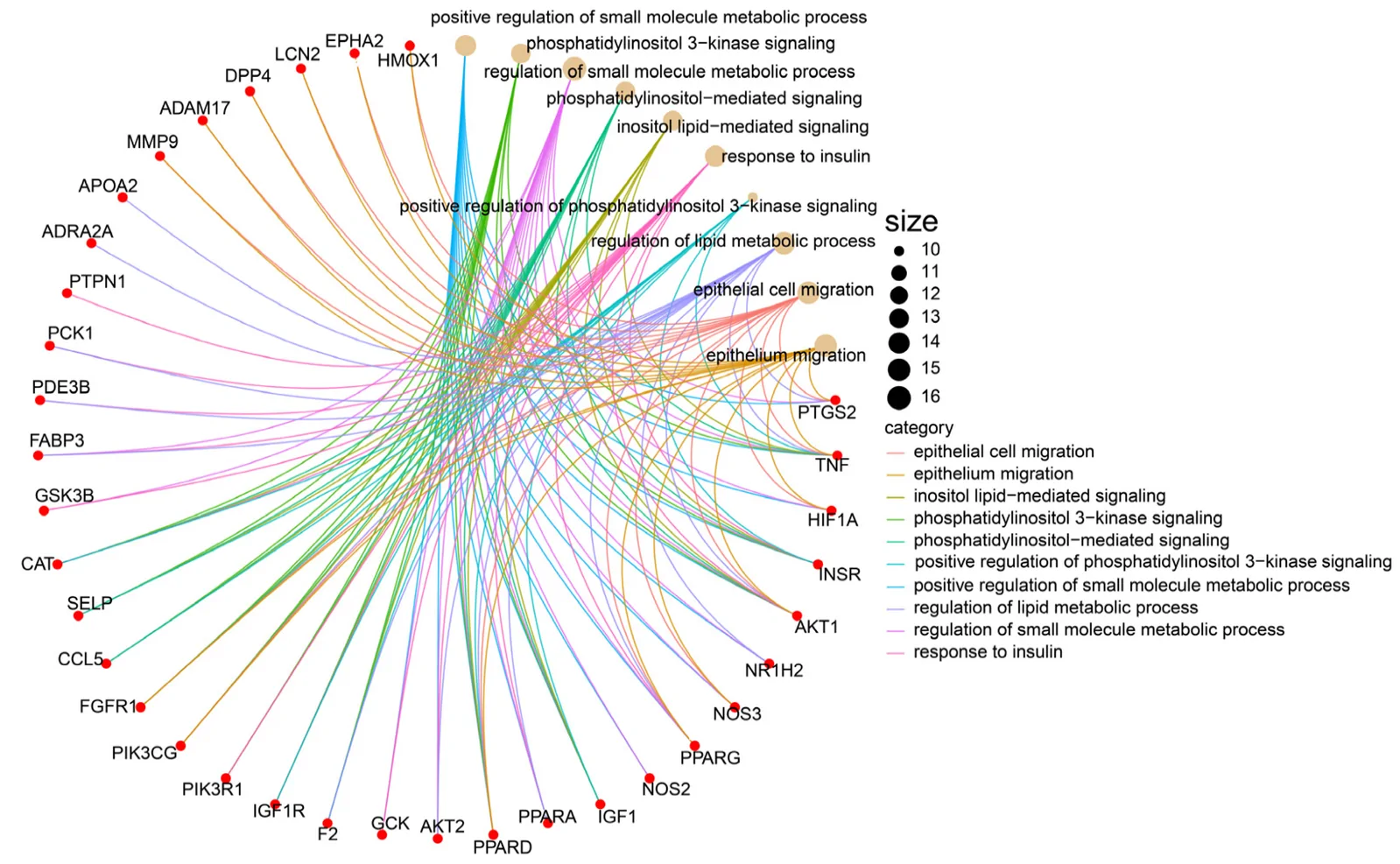
Gene-concept network of significantly enriched BP terms.

**Fig. (9):**
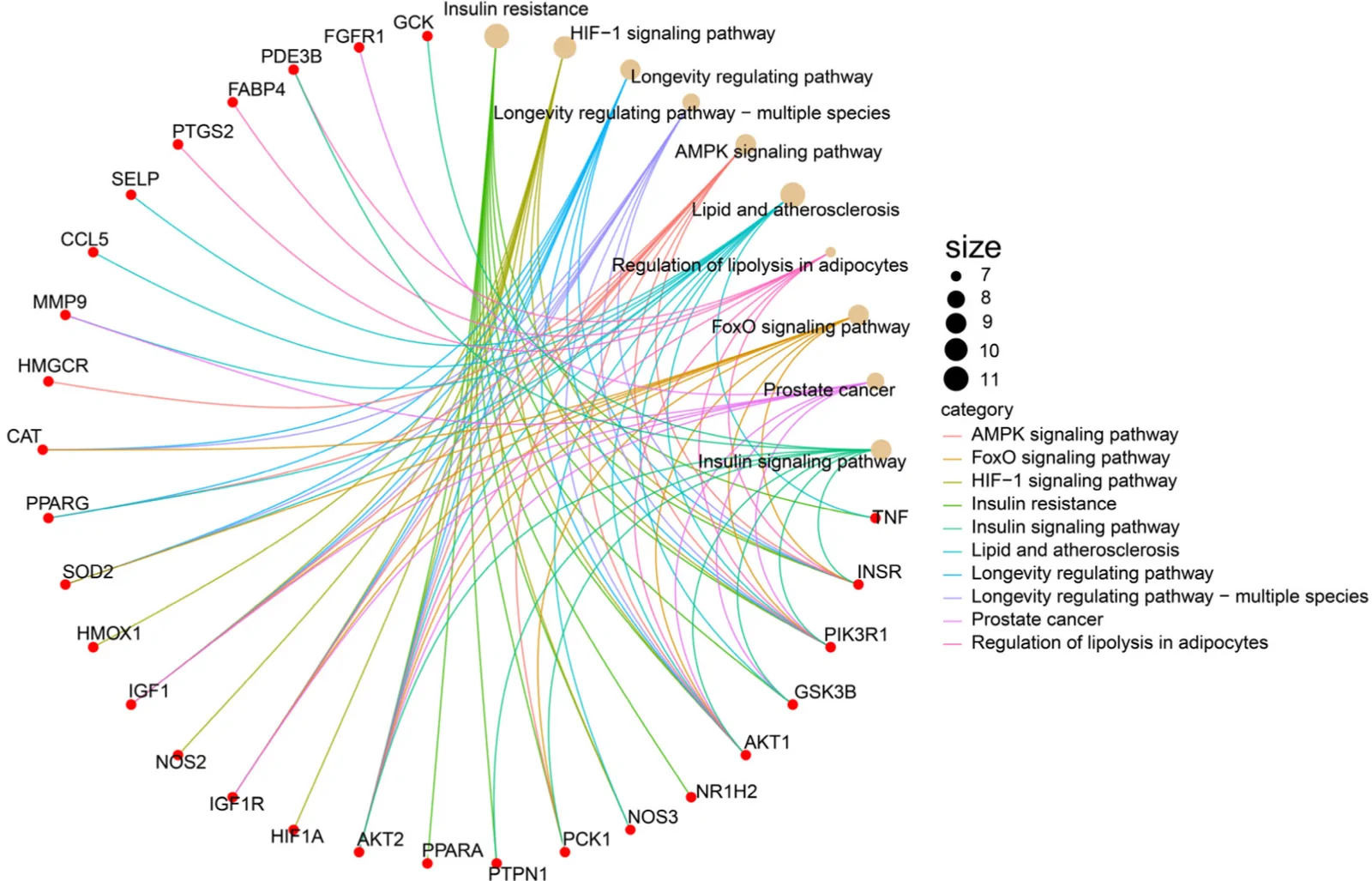
Gene-concept network of significantly enriched KEGG pathways.

**Fig. (11):**
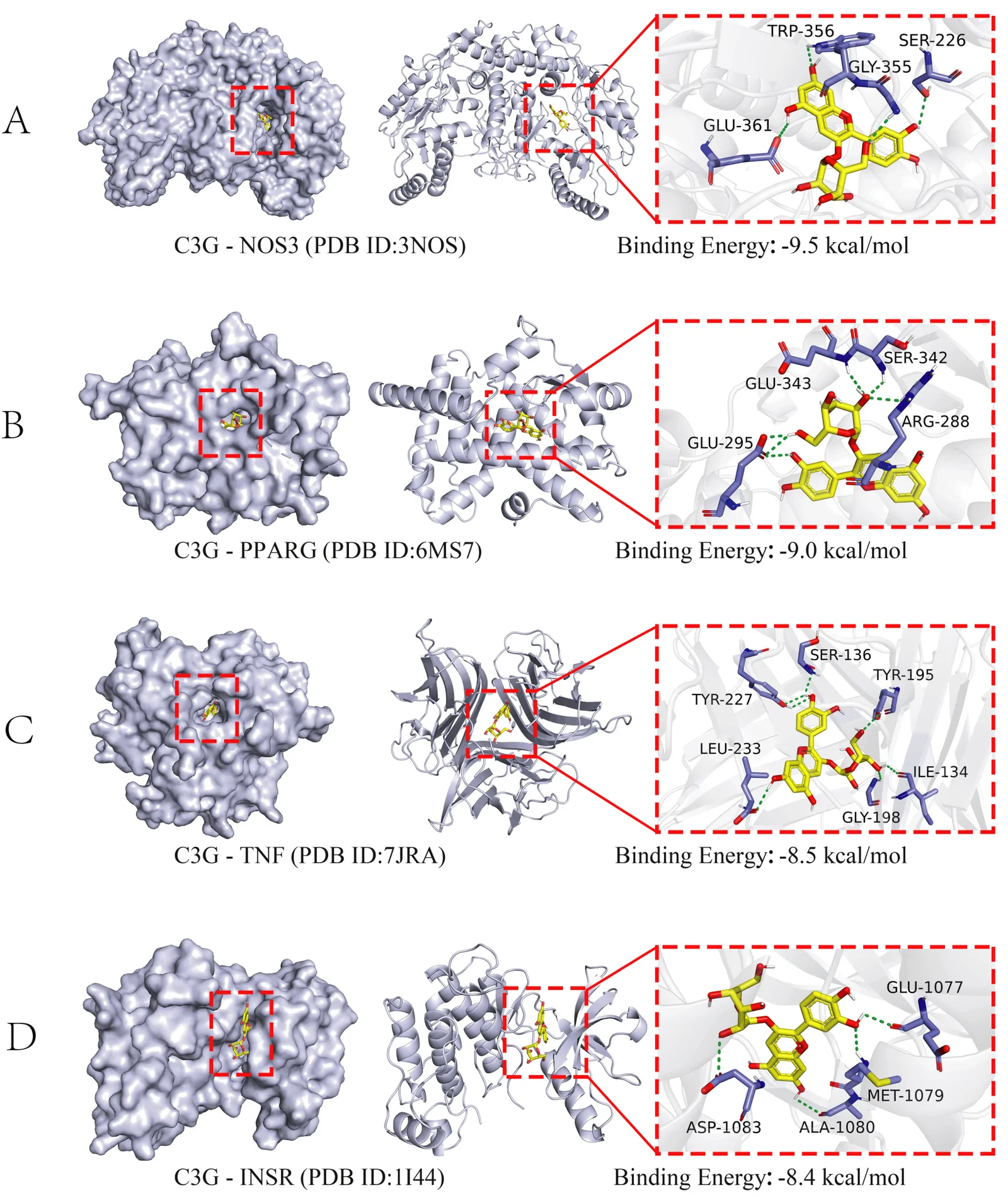
Predicted binding modes of C3G with core targets (NOS3, PPARG, TNF, INSR). (**A**) NOS3, (**B**) PPARG, (**C**)TNF, (**D**) INSR. In each subplot, C3G is shown as a yellow stick model, the predicted key hydrogen bonds are indicated by green dashed lines, and the key amino acid residues involved in the interactions are labeled.

**Table 1:** Molecular docking parameters of C3G with potential target proteins.

**Protein**	**Grid Center**			**Grid Size**		
	**X**	**Y**	**Z**	**X**	**Y**	**Z**
AKT1	6.000	0.000	19.000	26.000	26.000	24.000
TNF	0.000	0.000	-30.000	62.000	20.000	20.000
AKT2	15.029	-20.485	3.733	69.000	60.000	56.250
PPARG	46.641	8.915	18.607	48.000	56.000	66.000
IGF1	1.700	-3.700	-17.000	47.250	19.500	47.250
INSR	17.867	16.207	18.290	54.000	52.000	54.000
NOS3	20.890	10.430	22.720	15.000	15.000	15.000
CAT	20.911	61.008	58.352	90.000	114.000	86.000
ALB	22.401	4.064	3.320	27.000	26.000	20.000

**Note:** This table lists the coordinates and dimensions of the active site search grid defined for each protein in the molecular docking simulations. All parameters were set in AutoDock Vina. The units for both grid center (X, Y, Z) and grid size (X, Y, Z) are Angstroms (Å).

**Table 2:** **Key** targets and functional mechanisms.

**Target**	**Name**	**Degree**	**Functional Mechanism**	**References**
ALB	Albumin	43	Urinary albumin-to-creatinine ratio (UACR) is an early biomarker of diabetic nephropathy. Hyperglycemia induces albumin glycation to form advanced glycation end products (AGEs), which contribute to vascular injury.	[49]
AKT1	AKT Serine/Threonine Kinase 1	41	Core molecules of the insulin signaling pathway regulate GLUT4 translocation and glucose uptake. In type 2 diabetes mellitus (T2DM), reduced AKT1 phosphorylation levels contribute to insulin resistance.	[50]
TNF	Tumor Necrosis Factor	41	Adipose tissue-derived TNF-α impairs IRS-1 phosphorylation, directly contributing to insulin resistance.	[51]
PPARG	Peroxisome Proliferator-Activated Receptor Gamma	40	This mechanism regulates adipocyte differentiation and lipid storage, thereby improving peripheral insulin sensitivity. The Pro12Ala genetic variant is associated with an altered risk of type 2 diabetes mellitus (T2DM).	[52]
IGF1	Insulin-like Growth Factor 1	33	The structure is similar to that of insulin and partially activates the insulin signaling pathway *via* IGF1R, exerting a compensatory regulatory role under insulin-resistant conditions.	[53]
PTGS2	Prostaglandin-Endoperoxide Synthase 2	30	This pathway mediates prostaglandin synthesis and contributes to inflammatory damage in pancreatic β-cells. Hyperglycemia induces COX-2 overexpression, thereby promoting β-cell apoptosis.	[54]
PPARA	Peroxisome Proliferator-Activated Receptor Alpha	29	It promotes fatty acid oxidation by activating fatty acid oxidation-related genes (*e.g.*, CPT1A), thereby reducing hepatic lipid accumulation (lipotoxicity), and ameliorates insulin resistance by suppressing inflammatory cytokines (*e.g.*, TNF-α).	[55]
MMP9	Matrix Metalloproteinase 9	29	MMP-9 degrades the extracellular matrix, promoting diabetic retinopathy and atherosclerosis, and its serum levels positively correlate with vascular complication severity.	[56]
HIF1A	Hypoxia-Inducible Factor 1 Alpha	29	Chronic hyperglycemia promotes tissue hypoxia, leading to HIF-1α-driven oxidative stress and fibrosis.	[57]

## Data Availability

The datasets used and/or analyzed during the current study are available from the corresponding author upon reasonable request.

## LIST OF ABBREVIATIONS

C3G: Cyanidin-3-O-Glucoside
T2DM: Type 2 Diabetes Mellitus
PPI: Protein-Protein Interaction
GO: Gene Ontology
BP: Biological Processes
CC: Cellular Components
MF: Molecular Function
KEGG: Kyoto Encyclopedia of Genes and Genomes
TCMSP: Traditional Chinese Medicine Systems Pharmacology Database and Analysis Platform
TTD: Therapeutic Target Database
OMIM: Online Mendelian Inheritance in Man
RMSD: Root Mean Square Deviation
RMSF: Root Mean Square Fluctuation
Rg: Radius of Gyration
ALB: Albumin
Hnum: Hydrogen Number
AKT1: AKT Serine/Threonine Kinase 1
AKT2: AKT Serine/Threonine Kinase 2
TNF: Tumor Necrosis Factor
PPARG: Peroxisome Proliferator-Activated Receptor Gamma
IGF1: Insulin-like Growth Factor 1
PTGS2: Prostaglandin-Endoperoxide Synthase 2
PPARA: Peroxisome Proliferator-Activated Receptor Alpha
MMP9: Matrix Metalloproteinase 9
HIF1A: Hypoxia-Inducible Factor 1 Alpha
INSR: Insulin Receptor
NOS3: Nitric Oxide Synthase 3
CAT: Catalase
